# Di-μ-adipato-κ^4^ *O* ^1^:*O* ^6^-bis­{aqua­[5,6-di­phenyl-3-(pyridin-2-yl)-1,2,4-triazine-κ^2^ *N* ^2^,*N* ^3^]copper(II)}

**DOI:** 10.1107/S1600536812025676

**Published:** 2012-06-13

**Authors:** Wei Xu, Jin–Li Qi

**Affiliations:** a Center of Applied Solid State Chemistry Research, Ningbo University, Ningbo 315211, People’s Republic of China

## Abstract

In the centrosymmetric binuclear title complex, [Cu_2_(C_6_H_8_O_4_)_2_(C_20_H_14_N_4_)_2_(H_2_O)_2_] or [Cu_2_(*PDPT*)_2_(C_6_H_8_O_4_)_2_(H_2_O)_2_] (*PDPT* = 3-(2-pyrid­yl)-5,6-diphenyl-1,2,4-triazine, the Cu atom displays a distorted square-pyramidal coordination environment with the basal plane occupied by two *PDPT* N atoms and two O atoms from different adipate dianions while a water mol­ecule is located at the apical position. Of the two water H atoms, one participates in an intra­molecular hydrogen bond whereas the second participates in an inter­molecular hydrogen bond, which leads to the formation of a chain along [010].

## Related literature

For the biological activity and applications of triazines, see: Garcia *et al.* (1995 ▶); Mashaly *et al.* (1999 ▶); Croot & Hunter (2000 ▶); Soudi *et al.* (2005 ▶); Kawamichi *et al.* (2009 ▶).
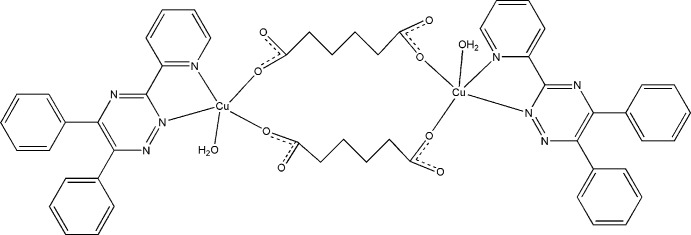


## Experimental

### 

#### Crystal data

[Cu_2_(C_6_H_8_O_4_)_2_(C_20_H_14_N_4_)_2_(H_2_O)_2_]
*M* *_r_* = 1072.08
Triclinic, 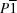

*a* = 9.4825 (19) Å
*b* = 10.616 (2) Å
*c* = 13.080 (3) Å
α = 78.96 (3)°
β = 68.76 (3)°
γ = 76.85 (3)°
*V* = 1186.4 (5) Å^3^
*Z* = 1
Mo *K*α radiation
μ = 0.97 mm^−1^
*T* = 295 K
0.30 × 0.19 × 0.11 mm

#### Data collection

Rigaku R-AXIS RAPID CCD diffractometer
Absorption correction: multi-scan (*ABSCOR*; Higashi, 1995 ▶) *T*_min_ = 0.756, *T*_max_ = 0.863
11646 measured reflections
5295 independent reflections
3207 reflections with *I* > 2σ(*I*)
*R*_int_ = 0.061

#### Refinement

*R*[*F* ^2^ > 2σ(*F* ^2^)] = 0.057
*wR*(*F* ^2^) = 0.135
*S* = 1.02
5295 reflections
333 parameters
3 restraints
H atoms treated by a mixture of independent and constrained refinement
Δρ_max_ = 0.61 e Å^−3^
Δρ_min_ = −0.51 e Å^−3^

### 

Data collection: *RAPID-AUTO* (Rigaku, 1998 ▶); cell refinement: *RAPID-AUTO*; data reduction: *CrystalStructure* (Rigaku/MSC, 2004 ▶); program(s) used to solve structure: *SHELXS97* (Sheldrick, 2008 ▶); program(s) used to refine structure: *SHELXL97* (Sheldrick, 2008 ▶); molecular graphics: *SHELXTL* (Sheldrick, 2008 ▶); software used to prepare material for publication: *SHELXL97*.

## Supplementary Material

Crystal structure: contains datablock(s) global, I. DOI: 10.1107/S1600536812025676/rk2363sup1.cif

Structure factors: contains datablock(s) I. DOI: 10.1107/S1600536812025676/rk2363Isup2.hkl

Additional supplementary materials:  crystallographic information; 3D view; checkCIF report

## Figures and Tables

**Table 1:** Hydrogen-bond geometry (Å, °)

*D*—H⋯*A*	*D*—H	H⋯*A*	*D*⋯*A*	*D*—H⋯*A*
O5—H5*A*⋯O3^i^	0.83	1.92	2.721 (3)	161
O5—H5*B*⋯O1^ii^	0.83	2.06	2.878 (4)	167

Symmetry codes: (i) 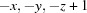; (ii) 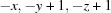.

**Fig. 1.**
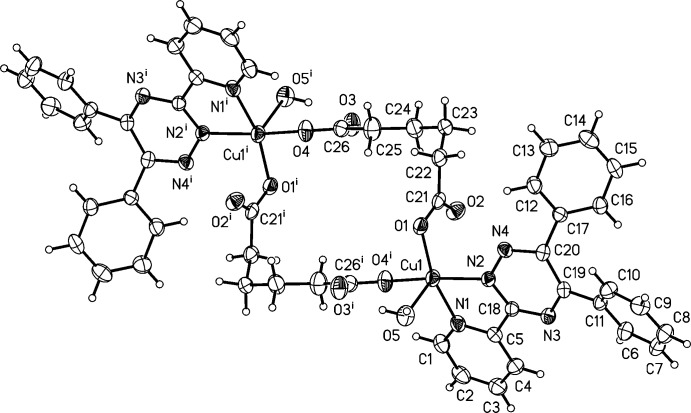
The binuclear structure of title compound with the atom numbering scheme. Displacement ellipsoids are drawn at 40% probability level. H atoms are presented as a small spheres of arbitrary radius. Symmetry code: (i) -x, -y, -z+1.Di-µ-adipato-κ4O1:O5-bis{aqua[5,6-diphenyl- 3-(pyridin-2-yl)-1,2,4-triazine-κ2N2,N3]copper(II)}

**Fig. 2.**
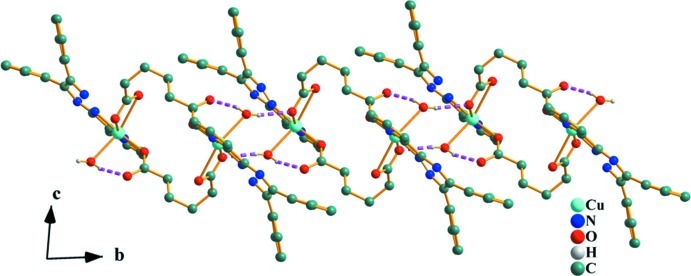
One dimensional chain through hydrogen bond along [0 1 0]. The C—H bonds omitted for clarity.

## supplementary crystallographic information

### Comment

Research on coordination chemistry of triazine–derived ligands has progressed very rapidly during the past two decades (Kawamichi *et al.*, 2009). The 1,2,4–triazine compounds are well–known in natural materials and show intersting biological, pharmacological and medicinal properties (Garcia *et al.*, 1995). The 3–(2–pyridyl)–5,6–diphenyl–1,2,4–triazine (*PDPT*) represents a principal class of *N*–donor heterocyclic ligands that exhibit interesting pharmacological properties such as blood platelet aggregation inhibition, significant activity towards leukemia and ovarian cancer, and anti–HIV activity (Mashaly *et al.*, 1999; Soudi *et al.*, 2005). It also has been widely used as an sensitive reagent for the determination of Fe(II) by spectrophotometric methods, in natural and waste water (Croot & Hunter, 2000). The title complex, was recently prepared and its crystal structure is reported here.

The title compound crystal structure is composed of centrosymmetric binuclear [Cu_2_(H_2_O)_2_(*PDPT*)_2_(C_6_H_8_O_4_)_2_] complex molecule (Fig. 1). The dinuclear complex molecules are centered at the crystallographic 2*e* positions. Each Cu atom is coordinated by two N atoms of the chelating *PDPT* ligand and three O atoms of one H_2_O molecule and two bis–monodentate adipato ligands to form a slightly distorted square–pyramidal coordination with H_2_O molecule located at the apical position (d(Cu–N) = 2.028 (3)Å, 2.029 (3)Å, the basal d(Cu–O) = 1.921 (3)Å, 1.961 (3)Å, the axial d(Cu–O) = 2.375 (3)Å). Through the adipato ligands, the square–pyramidally coordinated Cu atoms are linked to form centrosymmetric dinuclear. As expected, the Cu atom is slightly shifted toward the apical water O atom by 0.026 (2)Å from the least–squares plane defined by the four equatorial coordinating atoms. The triazine ring adopts a slight twist conformation. The dihedral angle between the two phenyl rings is 61.9 (2)°.

As shown in the Fig. 2 and Table 1, within the crystal structure, the water molecule O5 forms a strong intramolecular hydrogen bond to the uncoordinated carboxyl O3^i^ with O5···O3^i^ = 2.721 (3)Å and angle O5—H5A···O3^i^ = 161°. Moreover, it forms an intermolecular hydrogen bond to the coordinated carboxyl O1^ii^ atoms with O5···O1^ii^ = 2.878 (4)Å and angle O5—H5B···O1^ii^ = 167° to connect the dinuclear complexes along the [0 1 0] direction. Symmetry codes: (i) -*x*, -*y*, -*z*+1; (ii) -*x*, -*y*+1, -*z*+1.

### Experimental

Addition of 2.0 mL (1.0 *M*) NaOH to a stirred aqueous of 0.172 g (1.0 mmol) CuCl_2_^.^2H_2_O in 5.0 mL H_2_O yield a blue precipitate, which was then separated by centrifugation, followed by washing with double–distilled water until no detectable Cl^-^ anions in supernatant. The precipitate was added to a stirred ethanolic aqueous solution of 0.146 g (1.0 mmol) adipic acid in 20 mL *Et*OH/H_2_O (v:v = 1: 1). To the resulting suspension was added 0.310 g (1.0 mmol) 3–(2–pyridyl)–5,6–diphenyl–1,2,4–triazine (*PDPT*). The mixture was further stirred for approximately 15 min and the insoluble solid was filtered off. The filtrate (pH = 6.5) was allowed to stand at room temperature. Slow evaporation for two weeks afforded a small amount of brown crystals (yield 58% based on the initial CuCl_2_^.^2H_2_O input).

### Refinement

All H atoms bound to C were position geometrically and refined as riding, with C—H = 0.93Å and 0.97Å with *U*_iso_(H) = 1.2*U*_eq_(C). The H atoms attached to O were located in difference Fourier maps and refined freely with *U*_iso_(H) = 1.5*U*_eq_(O).

### Crystal data

**Table:**

[Cu_2_(C_6_H_8_O_4_)_2_(C_20_H_14_N_4_)_2_(H_2_O)_2_]	*Z* = 1
*M**_r_* = 1072.08	*F*(000) = 554
Triclinic, *P*1	*D*_x_ = 1.501 Mg m^−^^3^
Hall symbol: -P 1	Mo *K*α radiation, λ = 0.71073 Å
*a* = 9.4825 (19) Å	Cell parameters from 8025 reflections
*b* = 10.616 (2) Å	θ = 3.3–27.5°
*c* = 13.080 (3) Å	µ = 0.97 mm^−^^1^
α = 78.96 (3)°	*T* = 295 K
β = 68.76 (3)°	Plate, brown
γ = 76.85 (3)°	0.30 × 0.19 × 0.11 mm
*V* = 1186.4 (5) Å^3^	

### Data collection

**Table:**

Rigaku R-AXIS RAPID CCD diffractometer	5295 independent reflections
Radiation source: fine-focus sealed tube	3207 reflections with *I* > 2σ(*I*)
Graphite monochromator	*R*_int_ = 0.061
ω–scans	θ_max_ = 27.5°, θ_min_ = 3.3°
Absorption correction: multi-scan (*ABSCOR*; Higashi, 1995)	*h* = −12→11
*T*_min_ = 0.756, *T*_max_ = 0.863	*k* = −13→13
11646 measured reflections	*l* = −16→16

### Refinement

**Table:**

Refinement on *F*^2^	Primary atom site location: structure-invariant direct methods
Least-squares matrix: full	Secondary atom site location: difference Fourier map
*R*[*F*^2^ > 2σ(*F*^2^)] = 0.057	Hydrogen site location: inferred from neighbouring sites
*wR*(*F*^2^) = 0.135	H atoms treated by a mixture of independent and constrained refinement
*S* = 1.02	*w* = 1/[σ^2^(*F*_o_^2^) + (0.0516*P*)^2^ + 0.8435*P*] where *P* = (*F*_o_^2^ + 2*F*_c_^2^)/3
5295 reflections	(Δ/σ)_max_ < 0.001
333 parameters	Δρ_max_ = 0.61 e Å^−^^3^
3 restraints	Δρ_min_ = −0.51 e Å^−^^3^

### Special details

**Table:**

Geometry. All s.u.'s (except the s.u. in the dihedral angle between two l.s. planes) are estimated using the full covariance matrix. The cell s.u.'s are taken into account individually in the estimation of s.u.'s in distances, angles and torsion angles; correlations between s.u.'s in cell parameters are only used when they are defined by crystal symmetry. An approximate (isotropic) treatment of cell s.u.'s is used for estimating s.u.'s involving l.s. planes.
Refinement. Refinement of *F*^2^ against ALL reflections. The weighted *R*–factor w*R* and goodness of fit *S* are based on *F*^2^, conventional *R*–factors *R* are based on *F*, with *F* set to zero for negative *F*^2^. The threshold expression of *F*^2^ > 2σ(*F*^2^) is used only for calculating *R*–factors(gt) etc. and is not relevant to the choice of reflections for refinement. *R*–factors based on *F*^2^ are statistically about twice as large as those based on *F*, and *R*–factors based on ALL data will be even larger.

### Fractional atomic coordinates and isotropic or equivalent isotropic displacement parameters (Å^2^)

**Table:**

	*x*	*y*	*z*	*U*_iso_*/*U*_eq_	
Cu1	0.24237 (6)	0.28612 (5)	0.46540 (4)	0.03879 (17)	
N1	0.4452 (4)	0.2512 (3)	0.4938 (3)	0.0374 (8)	
N2	0.3453 (3)	0.4301 (3)	0.3587 (3)	0.0333 (7)	
N3	0.5595 (3)	0.5336 (3)	0.3071 (3)	0.0343 (7)	
N4	0.3041 (3)	0.4987 (3)	0.2743 (3)	0.0355 (7)	
O1	0.0833 (3)	0.3122 (3)	0.3970 (2)	0.0390 (6)	
O2	0.2814 (3)	0.1907 (3)	0.2883 (3)	0.0488 (7)	
O3	0.0418 (3)	−0.2164 (3)	0.3245 (3)	0.0570 (8)	
O4	−0.1845 (3)	−0.1321 (3)	0.4379 (3)	0.0488 (8)	
O5	0.0969 (3)	0.4299 (3)	0.5999 (3)	0.0495 (8)	
H5A	0.042 (4)	0.375 (3)	0.634 (4)	0.074*	
H5B	0.040 (4)	0.501 (2)	0.593 (4)	0.074*	
C1	0.4946 (5)	0.1514 (4)	0.5589 (3)	0.0436 (10)	
H1A	0.4325	0.0892	0.5955	0.052*	
C2	0.6347 (5)	0.1373 (4)	0.5739 (3)	0.0460 (11)	
H2A	0.6684	0.0647	0.6170	0.055*	
C3	0.7228 (5)	0.2320 (4)	0.5244 (4)	0.0471 (11)	
H3A	0.8152	0.2262	0.5362	0.057*	
C4	0.6740 (4)	0.3366 (4)	0.4566 (3)	0.0404 (9)	
H4A	0.7322	0.4020	0.4221	0.048*	
C5	0.5364 (4)	0.3401 (4)	0.4422 (3)	0.0333 (8)	
C6	0.7323 (4)	0.7259 (4)	0.1838 (4)	0.0414 (10)	
H6A	0.7876	0.6483	0.2068	0.050*	
C7	0.7968 (5)	0.8365 (4)	0.1501 (4)	0.0495 (11)	
H7A	0.8964	0.8330	0.1486	0.059*	
C8	0.7152 (5)	0.9530 (4)	0.1183 (4)	0.0520 (12)	
H8A	0.7591	1.0279	0.0959	0.062*	
C9	0.5676 (5)	0.9572 (4)	0.1200 (4)	0.0497 (11)	
H9A	0.5117	1.0358	0.0996	0.060*	
C10	0.5033 (5)	0.8481 (4)	0.1509 (4)	0.0419 (10)	
H10A	0.4046	0.8525	0.1502	0.050*	
C11	0.5839 (4)	0.7290 (3)	0.1840 (3)	0.0331 (8)	
C12	0.2046 (4)	0.6241 (4)	0.1017 (4)	0.0423 (10)	
H12A	0.1287	0.6023	0.1676	0.051*	
C13	0.1724 (5)	0.6510 (4)	0.0040 (4)	0.0492 (11)	
H13A	0.0761	0.6445	0.0043	0.059*	
C14	0.2816 (5)	0.6872 (4)	−0.0934 (4)	0.0510 (11)	
H14A	0.2587	0.7071	−0.1586	0.061*	
C15	0.4258 (5)	0.6942 (4)	−0.0942 (3)	0.0444 (10)	
H15A	0.4999	0.7191	−0.1601	0.053*	
C16	0.4600 (5)	0.6642 (4)	0.0026 (3)	0.0379 (9)	
H16A	0.5579	0.6675	0.0011	0.045*	
C17	0.3498 (4)	0.6293 (3)	0.1022 (3)	0.0323 (8)	
C18	0.4794 (4)	0.4412 (3)	0.3645 (3)	0.0308 (8)	
C19	0.5105 (4)	0.6140 (3)	0.2307 (3)	0.0320 (8)	
C20	0.3880 (4)	0.5838 (3)	0.2046 (3)	0.0322 (8)	
C21	0.1506 (4)	0.2554 (4)	0.3093 (3)	0.0363 (9)	
C22	0.0629 (5)	0.2733 (4)	0.2294 (4)	0.0424 (10)	
H22A	−0.0456	0.2783	0.2717	0.051*	
H22B	0.0762	0.3559	0.1834	0.051*	
C23	0.1097 (5)	0.1676 (4)	0.1553 (3)	0.0441 (10)	
H23A	0.2148	0.1694	0.1071	0.053*	
H23B	0.0456	0.1878	0.1090	0.053*	
C24	0.0991 (5)	0.0304 (4)	0.2136 (4)	0.0525 (11)	
H24A	0.1160	−0.0266	0.1590	0.063*	
H24B	0.1818	0.0018	0.2443	0.063*	
C25	−0.0463 (5)	0.0133 (4)	0.3027 (4)	0.0595 (13)	
H25A	−0.1292	0.0445	0.2724	0.071*	
H25B	−0.0616	0.0685	0.3583	0.071*	
C26	−0.0602 (5)	−0.1238 (4)	0.3594 (3)	0.0393 (9)	

### Atomic displacement parameters (Å^2^)

**Table:**

	*U*^11^	*U*^22^	*U*^33^	*U*^12^	*U*^13^	*U*^23^
Cu1	0.0376 (3)	0.0390 (3)	0.0392 (3)	−0.0141 (2)	−0.0134 (2)	0.0069 (2)
N1	0.0387 (18)	0.0370 (18)	0.034 (2)	−0.0073 (14)	−0.0125 (15)	0.0035 (15)
N2	0.0339 (16)	0.0338 (17)	0.0325 (19)	−0.0103 (13)	−0.0124 (14)	0.0033 (14)
N3	0.0330 (17)	0.0318 (17)	0.036 (2)	−0.0066 (13)	−0.0103 (14)	−0.0015 (14)
N4	0.0364 (17)	0.0380 (18)	0.0308 (19)	−0.0067 (14)	−0.0130 (14)	0.0026 (14)
O1	0.0372 (14)	0.0379 (15)	0.0414 (18)	−0.0135 (12)	−0.0097 (13)	−0.0023 (13)
O2	0.0369 (16)	0.0528 (18)	0.058 (2)	−0.0044 (14)	−0.0162 (14)	−0.0125 (15)
O3	0.0568 (19)	0.0408 (17)	0.061 (2)	−0.0117 (15)	−0.0073 (16)	0.0011 (16)
O4	0.0459 (17)	0.0403 (16)	0.050 (2)	−0.0119 (13)	−0.0076 (15)	0.0081 (14)
O5	0.0481 (18)	0.0387 (16)	0.056 (2)	−0.0060 (13)	−0.0129 (16)	−0.0035 (15)
C1	0.045 (2)	0.041 (2)	0.037 (3)	−0.0053 (18)	−0.0106 (19)	0.0029 (19)
C2	0.045 (2)	0.050 (3)	0.035 (3)	0.007 (2)	−0.016 (2)	−0.001 (2)
C3	0.034 (2)	0.062 (3)	0.044 (3)	0.000 (2)	−0.015 (2)	−0.010 (2)
C4	0.038 (2)	0.041 (2)	0.043 (3)	−0.0060 (18)	−0.0153 (19)	−0.0050 (19)
C5	0.035 (2)	0.034 (2)	0.029 (2)	−0.0043 (16)	−0.0095 (17)	−0.0043 (16)
C6	0.037 (2)	0.036 (2)	0.048 (3)	−0.0079 (17)	−0.0110 (19)	−0.0018 (19)
C7	0.045 (2)	0.046 (2)	0.060 (3)	−0.020 (2)	−0.012 (2)	−0.007 (2)
C8	0.069 (3)	0.037 (2)	0.055 (3)	−0.027 (2)	−0.018 (2)	0.001 (2)
C9	0.068 (3)	0.030 (2)	0.054 (3)	−0.009 (2)	−0.026 (2)	0.001 (2)
C10	0.046 (2)	0.033 (2)	0.050 (3)	−0.0080 (17)	−0.019 (2)	−0.0043 (19)
C11	0.037 (2)	0.0273 (18)	0.035 (2)	−0.0111 (15)	−0.0100 (17)	−0.0014 (16)
C12	0.035 (2)	0.050 (2)	0.037 (2)	−0.0076 (18)	−0.0116 (18)	0.0057 (19)
C13	0.042 (2)	0.058 (3)	0.047 (3)	−0.004 (2)	−0.021 (2)	0.001 (2)
C14	0.064 (3)	0.046 (3)	0.039 (3)	0.002 (2)	−0.023 (2)	0.000 (2)
C15	0.057 (3)	0.036 (2)	0.029 (2)	−0.0072 (19)	−0.002 (2)	−0.0014 (18)
C16	0.040 (2)	0.033 (2)	0.037 (2)	−0.0084 (16)	−0.0085 (18)	−0.0025 (17)
C17	0.041 (2)	0.0256 (18)	0.031 (2)	−0.0087 (15)	−0.0126 (17)	−0.0012 (16)
C18	0.0319 (19)	0.0302 (19)	0.029 (2)	−0.0081 (15)	−0.0077 (16)	−0.0034 (16)
C19	0.0287 (18)	0.0308 (19)	0.032 (2)	−0.0051 (15)	−0.0064 (16)	−0.0005 (16)
C20	0.0288 (18)	0.0264 (18)	0.037 (2)	−0.0041 (15)	−0.0071 (16)	−0.0027 (16)
C21	0.036 (2)	0.031 (2)	0.042 (3)	−0.0163 (17)	−0.0109 (18)	0.0036 (18)
C22	0.046 (2)	0.038 (2)	0.043 (3)	−0.0154 (18)	−0.017 (2)	0.0094 (19)
C23	0.049 (2)	0.052 (3)	0.034 (2)	−0.021 (2)	−0.016 (2)	0.008 (2)
C24	0.064 (3)	0.048 (3)	0.041 (3)	−0.020 (2)	−0.006 (2)	−0.007 (2)
C25	0.059 (3)	0.046 (3)	0.062 (4)	−0.013 (2)	−0.007 (3)	−0.002 (2)
C26	0.046 (2)	0.034 (2)	0.038 (3)	−0.0121 (18)	−0.014 (2)	−0.0003 (18)

### Geometric parameters (Å, º)

**Table:**

Cu1—O1	1.961 (3)	C8—C9	1.382 (6)
Cu1—O4^i^	1.921 (3)	C8—H8A	0.9300
Cu1—O5	2.375 (3)	C9—C10	1.356 (6)
Cu1—N1	2.029 (3)	C9—H9A	0.9300
Cu1—N2	2.028 (3)	C10—C11	1.401 (5)
N1—C1	1.336 (5)	C10—H10A	0.9300
N1—C5	1.341 (5)	C11—C19	1.463 (5)
N2—C18	1.333 (4)	C12—C13	1.382 (6)
N2—N4	1.334 (4)	C12—C17	1.394 (5)
N3—C18	1.321 (5)	C12—H12A	0.9300
N3—C19	1.332 (4)	C13—C14	1.371 (6)
N4—C20	1.329 (5)	C13—H13A	0.9300
O1—C21	1.283 (5)	C14—C15	1.382 (6)
O2—C21	1.235 (4)	C14—H14A	0.9300
O3—C26	1.236 (5)	C15—C16	1.380 (6)
O4—C26	1.260 (5)	C15—H15A	0.9300
O4—Cu1^i^	1.921 (3)	C16—C17	1.390 (5)
O5—H5A	0.829 (18)	C16—H16A	0.9300
O5—H5B	0.830 (18)	C17—C20	1.477 (5)
C1—C2	1.383 (5)	C19—C20	1.438 (5)
C1—H1A	0.9300	C21—C22	1.516 (5)
C2—C3	1.367 (6)	C22—C23	1.508 (6)
C2—H2A	0.9300	C22—H22A	0.9700
C3—C4	1.385 (6)	C22—H22B	0.9700
C3—H3A	0.9300	C23—C24	1.516 (6)
C4—C5	1.376 (5)	C23—H23A	0.9700
C4—H4A	0.9300	C23—H23B	0.9700
C5—C18	1.481 (5)	C24—C25	1.470 (6)
C6—C7	1.372 (6)	C24—H24A	0.9700
C6—C11	1.399 (5)	C24—H24B	0.9700
C6—H6A	0.9300	C25—C26	1.513 (6)
C7—C8	1.378 (6)	C25—H25A	0.9700
C7—H7A	0.9300	C25—H25B	0.9700
			
O1—Cu1—O5	94.47 (11)	C13—C12—H12A	119.7
O1—Cu1—N1	164.40 (13)	C17—C12—H12A	119.7
O1—Cu1—N2	92.22 (11)	C14—C13—C12	120.4 (4)
O4^i^—Cu1—O1	96.02 (12)	C14—C13—H13A	119.8
O4^i^—Cu1—O5	94.45 (12)	C12—C13—H13A	119.8
O4^i^—Cu1—N1	90.71 (12)	C13—C14—C15	119.8 (4)
O4^i^—Cu1—N2	168.98 (12)	C13—C14—H14A	120.1
N1—Cu1—O5	99.04 (12)	C15—C14—H14A	120.1
N2—Cu1—O5	92.19 (12)	C16—C15—C14	120.1 (4)
N2—Cu1—N1	79.55 (12)	C16—C15—H15A	120.0
C1—N1—C5	117.8 (3)	C14—C15—H15A	120.0
C1—N1—Cu1	126.9 (3)	C15—C16—C17	120.8 (4)
C5—N1—Cu1	115.3 (2)	C15—C16—H16A	119.6
C18—N2—N4	117.7 (3)	C17—C16—H16A	119.6
C18—N2—Cu1	115.4 (2)	C16—C17—C12	118.3 (3)
N4—N2—Cu1	126.0 (2)	C16—C17—C20	121.7 (3)
C18—N3—C19	117.8 (3)	C12—C17—C20	119.6 (3)
C20—N4—N2	120.5 (3)	N3—C18—N2	124.6 (3)
C21—O1—Cu1	104.0 (2)	N3—C18—C5	120.5 (3)
C26—O4—Cu1^i^	126.9 (3)	N2—C18—C5	114.9 (3)
Cu1—O5—H5A	90 (4)	N3—C19—C20	118.0 (3)
Cu1—O5—H5B	129 (4)	N3—C19—C11	115.9 (3)
H5A—O5—H5B	108 (3)	C20—C19—C11	126.0 (3)
N1—C1—C2	122.4 (4)	N4—C20—C19	118.7 (3)
N1—C1—H1A	118.8	N4—C20—C17	114.1 (3)
C2—C1—H1A	118.8	C19—C20—C17	127.0 (3)
C3—C2—C1	118.8 (4)	O2—C21—O1	122.7 (4)
C3—C2—H2A	120.6	O2—C21—C22	120.5 (4)
C1—C2—H2A	120.6	O1—C21—C22	116.8 (3)
C2—C3—C4	119.8 (4)	C23—C22—C21	115.3 (3)
C2—C3—H3A	120.1	C23—C22—H22A	108.5
C4—C3—H3A	120.1	C21—C22—H22A	108.5
C5—C4—C3	117.6 (4)	C23—C22—H22B	108.5
C5—C4—H4A	121.2	C21—C22—H22B	108.5
C3—C4—H4A	121.2	H22A—C22—H22B	107.5
N1—C5—C4	123.4 (3)	C22—C23—C24	115.8 (4)
N1—C5—C18	114.3 (3)	C22—C23—H23A	108.3
C4—C5—C18	122.2 (4)	C24—C23—H23A	108.3
C7—C6—C11	120.4 (4)	C22—C23—H23B	108.3
C7—C6—H6A	119.8	C24—C23—H23B	108.3
C11—C6—H6A	119.8	H23A—C23—H23B	107.4
C6—C7—C8	120.6 (4)	C25—C24—C23	116.0 (4)
C6—C7—H7A	119.7	C25—C24—H24A	108.3
C8—C7—H7A	119.7	C23—C24—H24A	108.3
C7—C8—C9	119.3 (4)	C25—C24—H24B	108.3
C7—C8—H8A	120.3	C23—C24—H24B	108.3
C9—C8—H8A	120.3	H24A—C24—H24B	107.4
C10—C9—C8	120.9 (4)	C24—C25—C26	116.6 (4)
C10—C9—H9A	119.6	C24—C25—H25A	108.2
C8—C9—H9A	119.6	C26—C25—H25A	108.2
C9—C10—C11	120.7 (4)	C24—C25—H25B	108.2
C9—C10—H10A	119.6	C26—C25—H25B	108.2
C11—C10—H10A	119.6	H25A—C25—H25B	107.3
C6—C11—C10	118.1 (3)	O3—C26—O4	125.7 (4)
C6—C11—C19	119.8 (3)	O3—C26—C25	120.3 (4)
C10—C11—C19	121.6 (3)	O4—C26—C25	113.9 (4)
C13—C12—C17	120.5 (4)		

Symmetry code: (i) −*x*, −*y*, −*z*+1.

### Hydrogen-bond geometry (Å, º)

**Table:**

*D*—H···*A*	*D*—H	H···*A*	*D*···*A*	*D*—H···*A*
O5—H5*A*···O3^i^	0.83	1.92	2.721 (3)	161
O5—H5*B*···O1^ii^	0.83	2.06	2.878 (4)	167

Symmetry codes: (i) −*x*, −*y*, −*z*+1; (ii) −*x*, −*y*+1, −*z*+1.
